# Seven-year performance of a clinical metagenomic next-generation sequencing test for diagnosis of central nervous system infections

**DOI:** 10.1038/s41591-024-03275-1

**Published:** 2024-11-12

**Authors:** Patrick Benoit, Noah Brazer, Mikael de Lorenzi-Tognon, Emily Kelly, Venice Servellita, Miriam Oseguera, Jenny Nguyen, Jack Tang, Charles Omura, Jessica Streithorst, Melissa Hillberg, Danielle Ingebrigtsen, Kelsey Zorn, Michael R. Wilson, Tim Blicharz, Amy P. Wong, Brian O’Donovan, Brad Murray, Steve Miller, Charles Y. Chiu

**Affiliations:** 1 Department of Laboratory Medicine, University of California, San Francisco, San Francisco, CA USA; 2 Department of Biochemistry and Biophysics, University of California, San Francisco, San Francisco, CA USA; 3 Weill Institute for Neurosciences, University of California, San Francisco, San Francisco, CA USA; 4 Department of Neurology, University of California, San Francisco, San Francisco, CA USA; 5 Delve Bio, Boston, MA USA; 6 Department of Medicine, University of California, San Francisco, San Francisco, CA USA; 7 Chan-Zuckerberg Biohub, San Francisco, CA USA

**Keywords:** Next-generation sequencing, Infectious diseases, Central nervous system infections, Translational research

## Abstract

Metagenomic next-generation sequencing (mNGS) of cerebrospinal fluid (CSF) is an agnostic method for broad-based diagnosis of central nervous system (CNS) infections. Here we analyzed the 7-year performance of clinical CSF mNGS testing of 4,828 samples from June 2016 to April 2023 performed by the University of California, San Francisco (UCSF) clinical microbiology laboratory. Overall, mNGS testing detected 797 organisms from 697 (14.4%) of 4,828 samples, consisting of 363 (45.5%) DNA viruses, 211 (26.4%) RNA viruses, 132 (16.6%) bacteria, 68 (8.5%) fungi and 23 (2.9%) parasites. We also extracted clinical and laboratory metadata from a subset of the samples (*n* = 1,164) from 1,053 UCSF patients. Among the 220 infectious diagnoses in this subset, 48 (21.8%) were identified by mNGS alone. The sensitivity, specificity and accuracy of mNGS testing for CNS infections were 63.1%, 99.6% and 92.9%, respectively. mNGS testing exhibited higher sensitivity (63.1%) than indirect serologic testing (28.8%) and direct detection testing from both CSF (45.9%) and non-CSF (15.0%) samples (*P* < 0.001 for all three comparisons). When only considering diagnoses made by CSF direct detection testing, the sensitivity of mNGS testing increased to 86%. These results justify the routine use of diagnostic mNGS testing for hospitalized patients with suspected CNS infection.

## Abstract (web summary)

Next-generation metagenomic testing increases the accuracy and sensitivity of the etiology of central nervous system infections in hospitalized patients.

## Main

Meningitis, encephalitis and/or myelitis associated with infections of the central nervous system (CNS) can cause severe and often life-threatening illness^1,2^. Timely diagnosis and treatment are paramount to improve clinical outcomes for these infections, and delays have been associated with increased morbidity and mortality^3,4^. A comprehensive infectious workup requires a combination of culture-based, serologic and nucleic acid amplification testing. However, it is estimated that the cause of meningoencephalitis remains unknown in approximately 50% of cases, which hinders clinical management and the initiation of appropriate and effective therapy^5,6^.

In recent years, clinical metagenomic next-generation sequencing (mNGS) has emerged as a comprehensive approach for infectious disease diagnosis, enabling simultaneous detection of a wide range of microorganisms, including bacteria, viruses, fungi and parasites, without targeting any specific pathogen a priori^7,8^. This agnostic, hypothesis-free method can be particularly useful in CNS infections for which the differential diagnosis is broad, with overlapping clinical manifestations, and for which cerebrospinal fluid (CSF) and brain biopsy tissue samples are often limited in volume and availability^6,9–13^.

The University of California, San Francisco (UCSF) CSF mNGS test, referred henceforth as ‘mNGS test/testing’, was developed in 2016 as a clinically validated DNA/RNA metagenomic sequencing assay performed by the Clinical Laboratory Improvement Amendments-certified UCSF clinical microbiology laboratory^14^. A prior prospective study demonstrated that this mNGS test can increase diagnostic yield and provide actionable information for CNS infections^6^. Here we sought to evaluate the clinical applicability of mNGS testing performed over 7 years as part of the diagnostic workup for a geographically broad population of patients with suspected but ‘difficult-to-diagnose’ CNS infection. Test performance was assessed across 4,828 samples, including a subset of 1,164 samples from 1,053 UCSF patients for whom clinical adjudication and retrospective chart review were performed.

## Results

### Longitudinal mNGS testing

**Fig. 1:**
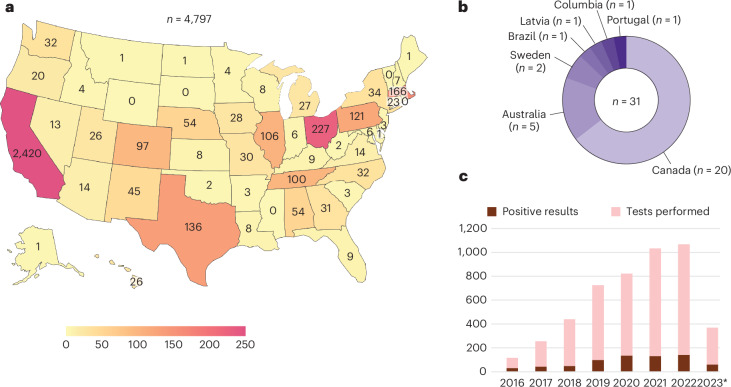
Distribution of tests ordered by year and geographic location. **a**,**b**, Distribution of tests ordered by state (**a**) and internationally (**b**). A total of 4,075 mNGS tests were performed from CSF samples collected from the United States, California being the most frequent state of origin (*n* = 2,420 samples). Reference laboratories such as Associated Regional and University Pathologists, Inc., Labcorp and Mayo Clinic (*n* = 722) receive tests from multiple states, so the location of individual samples cannot be tracked and thus are excluded from the figure. 14.8% (*n* = 715) of samples were sent from pediatric hospitals. **c**, Number of tests performed by year and number of positive results, excluding results that were reported possible or likely contaminants. *Data shown are samples analyzed up to April 2023. Source data

A total of 4,828 mNGS tests were performed from June 2016 to April 2023 (Table 1). The number of tests performed annually increased by nearly 10-fold from 116 in 2016 to 1,067 in 2022 (Fig. 1). Approximately 56% of patients were male; the mean age was 41.5 years, and children, defined as being younger than 18 years of age, comprised 24.2% of the cohort. Most mNGS tests (*n* = 4,075, 84.4%) were performed for US patients representing 46 different states, with 2,420 tests (50.1%) from California, 722 tests (15.0%) from regional or national reference laboratories in the United States and 31 tests (0.64%) from other countries (Fig. 1). The median turnaround times for UCSF and non-UCSF patients were 8.2 and 11.4 days, respectively, from sample collection to result (*P* < 0.0001), and 3.6 and 3.8 days, respectively, from start of sample processing in the laboratory to result (*P* < 0.0001). The longer turnaround times for non-UCSF patients are explained by delays resulting from the clinician decision to order mNGS testing, shipping of samples, and accessioning, aliquoting and batch testing of samples after receiving them in the laboratory.

**Table 1:** Patient and mNGS test characteristics associated with 4,828 CSF samples tested from 2016 to 2023

Patient or mNGS test characteristic		Number (%) of samples, turnaround time in days [IQR], or number (%) of detected organisms for all samples (*n* = 4,828 samples)	Number (%) of mNGS positive samples each year	Number (%) of samples, turnaround time in days [IQR], or number (%) of detected organisms for mNGS positive UCSF samples (*n* = 1,130 samples)	Number (%) of samples, turnaround time in days [IQR], or number (%) of detected organisms for mNGS positive non-UCSF samples (*n* = 3,698 samples)	Comparison between UCSF and non-UCSF samples
Sex						
	Male	2,558 (53.0%)				
	Female	2,038 (42.2%)				
	Other/not available	232 (4.8%)				
Age						
	<18	1,164 (24.1%)				
	18–65	2,638 (54.6%)				
	>65	1,003 (20.8%)				
	Not available	23 (0.5%)				
Year of CSF mNGS testing						
	2016	116 (2.4%)	31 (26.7%)			
	2017	256 (5.3%)	44 (17.2%)			
	2018	439 (9.1%)	50 (11.4%)			
	2019	726 (15%)	99 (13.6%)			
	2020	822 (17%)	136 (16.5%)			
	2021	1,032 (21.3%)	132 (12.8%)			
	2022	1,067 (22.1%)	141 (13.2%)			
	2023^a^	370 (7.6%)	61 (16.5%)			
QC metrics^b^						
	High human background DNA library	589 (12.2%)				
	High human background RNA library	79 (1.6%)				
	Low reads DNA library	124 (2.6%)				
	Low reads RNA library	255 (5.3%)				
	QC failure DNA library	52 (1.1%)				
	QC failure RNA library	38 (0.8%)				
Median turnaround time						
	Laboratory turnaround time in days [IQR]	3.7 [3.3, 5.0]		3.6 [3.3, 4.6]	3.8 [3.3, 5.2]	*P* < 0.0001^c^
	Total turnaround time in days [IQR]	10.5 [8.4, 14.2]		8.2 [6.3, 10.1]	11.4 [9.2, 15.1]	*P* < 0.0001^c^
Sequencing results^d^						
	Positive samples, excluding possible or likely contaminant	697 (14.4%)		183 (16.2%)	514 (13.9%)	*P* = 0.0547^e^
	Samples with positive subthreshold results	98 (2%)		32 (2.8%)	66 (1.8%)	*P* = 0.0289^e^
	Samples with single organism, possible contaminant	164 (3.4%)		23 (2%)	141 (3.8%)	*P* = 0.0039^e^
	Samples with multiple bacterial/fungal taxa, probable contaminant	348 (7.2%)		61 (5.4%)	287 (7.8%)	*P* = 0.0072^e^
Number (%) of detected organisms						
	All organisms	797 (100%)		222 (100%)	575 (100%)	
	Bacteria	132 (16.6%)		33 (14.9%)	99 (17.2%)	
	DNA virus	363 (45.5%)		103 (46.4%)	260 (45.2%)	
	RNA virus	211 (26.4%)		50 (22.5%)	161 (28%)	
	Fungi	68 (8.5%)		25 (11.2%)	43 (7.5%)	
	Parasite	23 (2.9%)		11 (5.0%)	12 (2.1%)	

IQR, interquartile range; QC, quality control.

^a^Data are up to April 2023.

^b^See Methods for a description of quality-control metrics.

^c^Comparison of turnaround times was done using the two-sided Mann-Whitney test.

^d^Percentages for each category are based on the total number of samples, as one sample can fit in more than one category (for example, concurrent positive detection of RNA virus and detection of multiple bacterial/fungal taxa suggesting contamination). See Methods for a description of the categories.

^e^Comparisons of the positivity rate between UCSF and non-UCSF samples were calculated using the two-sided Chi-squared test. All statistical tests were performed without adjustment for multiple comparisons.

We also evaluated quality control (QC) metrics associated with mNGS testing. High host background was more frequently observed in DNA (12.2%) than RNA (1.6%) libraries. This finding was attributed to higher efficiency of DNase treatment of RNA libraries in reducing background compared to antibody-based methylated DNA removal for DNA libraries^14^. RNA libraries were more difficult to amplify due to low amounts of input nucleic acid and/or degradation, resulting in low read counts of <5 million that were seen more frequently in RNA libraries (5.3%) than DNA libraries (2.6%). QC failure due to inadvertent errors in sample processing that required repeat testing was rarely observed (<1%).

One or more commensal and/or environmental organisms were detected in 512 (10.6%) of 4,828 samples. These results were all classified as negative after review by the laboratory director and were reported as possible (single taxon) or likely (multiple taxa) contaminants^6^. Contaminants were reported more often for non-UCSF compared to UCSF samples (7.8% versus 5.4%, *P* = 0.0072 and 3.8% versus 2%, *P* = 0.0039, for multiple taxa and a single organism, respectively), results that were attributed to differences in sample collection, handling and/or transport.

After excluding microorganisms reported as contaminants, 697 (14.4%) of 4,828 samples were positive for detection of a pathogen (Table 1 and Supplementary Dataset 1). The mean annual positivity rate was 16.0% ± 4.8% standard deviation and ranged from 11.4% to 27%. Notably, the positivity rate was 27% in 2016, the year during which nearly all patients had been enrolled in the prospective Precision Diagnosis of Acute Infectious Diseases (PDAID) study^6^. Of note, all PDAID participants met inclusion criteria of (i) hospitalization with an acute presentation and objective evidence of meningitis, encephalitis and/or myelitis within 2 weeks of CSF sampling and (ii) lack of a diagnosis at time of enrollment. The positivity rate in UCSF samples (16.2%) was higher than in non-UCSF samples (13.9%) (*P* = 0.0547). This difference can be explained in part by the higher rate of subthreshold results (2.8% versus 1.8%, P = 0.0289) in UCSF patients who were reported as positives, defined as detection of reads to presumptive pathogens at levels below pre-established thresholds^14^. For UCSF patients, the laboratory director was able to review the patient electronic medical records, hold discussions with the clinical teams caring for the patient and examine results of surveys taken at the time mNGS testing was ordered to determine if a subthreshold result was consistent with clinical findings, thus reporting it as a positive result. Pathogens detected at subthreshold levels included *Coccidioides sp*. in 15 (93.4%) of 16 samples, *Mycobacterium tuberculosis* in 12 (92.3%) of 13 samples, *Balamuthia mandrillaris* in 2 (66%) of 3 samples, *Histoplasma capsulatum* in 2 (50%) of 4 samples, West Nile virus in 8 (28.6%) of 28 samples and Powassan virus in 3 (21.4%) of 14 samples (Supplementary Dataset 1). Most subthreshold detections by mNGS testing were able to be confirmed by orthogonal testing from another method, such as serology or PCR.

**Fig. 2:**
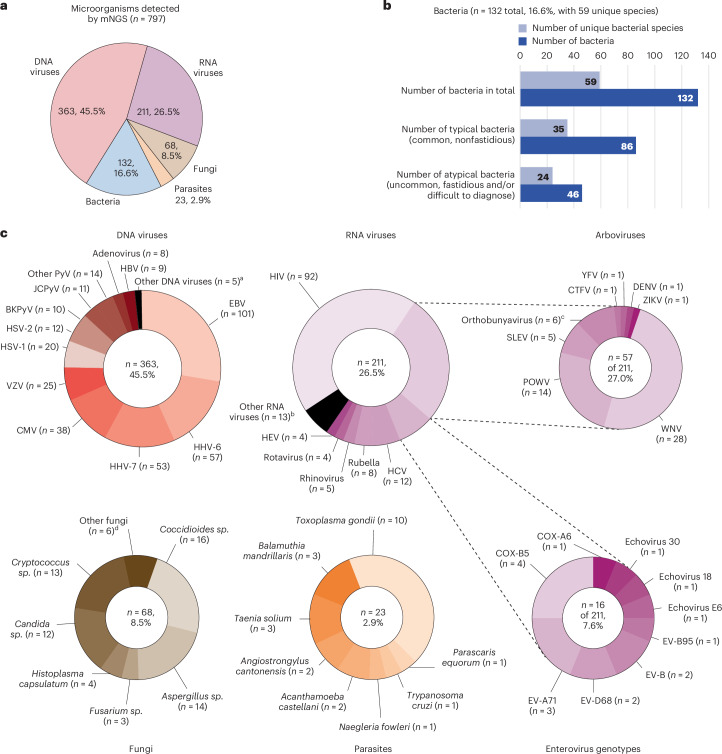
Summary of positive results by mNGS testing (*n* = 4,828). **a**, Number and types of organisms detected by mNGS testing. **b**, Detected bacterial species (total and unique), including those that are typical (nonfastidious) and atypical (uncommon, fastidious and/or difficult to diagnose). **c**, Detected DNA viruses, RNA viruses (including arboviruses and enteroviruses), fungi and parasites. ^a^Other DNA viruses detected included human parvovirus 4 (*n* = 1), human parvovirus B19 (*n* = 4) and human herpesvirus 8 (*n* = 1). ^b^Other RNA viruses detected included lymphocytic choriomeningitis virus (*n* = 3), astrovirus (*n* = 2), calicivirus (*n* = 2), coronavirus 229E (*n* = 2), SARS-CoV-2 (*n* = 1), human T cell lymphotropic virus (*n* = 1), human parechovirus (*n* = 1) and measles virus (*n* = 1). ^c^Orthobunyaviruses detected included Cache Valley virus (*n* = 3), Jamestown Canyon virus (*n* = −1), La Crosse virus (*n* = 1) and Potosi virus (*n* = 1). Potosi virus is a novel species in this genus, not reported before as causing disease in humans^16^. ^d^Other fungi detected included *Alternaria sp*. (*n* = 2), *Mucorales sp*. (*n* = 2), *Epicoccum sp*. (*n* = 1) and *Sporothrix schenkii* (*n* = 1). BKPyV, BK polyomavirus or human polyomavirus 1; CMV, cytomegalovirus; COX, coxsackievirus; CTFV, Colorado tick fever virus; DENV, dengue virus; EBV, Epstein-Barr virus; EV, enterovirus; HBV, hepatitis B virus; HCV, hepatitis C virus; HEV, hepatitis E virus; HHV-6, human herpesvirus 6; HHV-7, human herpesvirus 7; HSV, herpes simplex virus; HIV, human immunodeficiency virus; HTLV-2, human T cell lymphotropic virus 2; JCPyV, JC polyomavirus or human polyomavirus 2; NAAT, nucleic acid amplification testing; POWV, Powassan virus; SLEV, St. Louis encephalitis virus; VZV, varicella-zoster virus; WNV, West Nile virus; YFV, yellow fever virus; ZIKV, Zika virus. Source data

Among 697 mNGS-positive samples, 797 organisms were identified. DNA viruses were most frequently detected (*n* = 363, 45.5%), followed by RNA viruses (*n* = 211, 26.4%), bacteria (*n* = 132, 16.6%), fungi (*n* = 68, 8.5%) and parasites (*n* = 23, 2.9%) (Fig. 2a). The assay identified 86 nonfastidious bacterial pathogens representing 35 unique species and corresponding to bacteria that traditional culture methods can readily detect **(**Fig. 2b and Supplementary Table 1). The assay also detected bacterial CNS pathogens representing 24 unique species that are difficult and/or slow to grow in culture, including *Mycobacterium tuberculosis* (*n* = 13), *Nocardia farcinica* (*n* = 3), *Borrelia burgdorferi* (*n* = 2), *Leptospira borgpetersenii* (*n* = 1), *Borrelia miyamotoi* (*n* = 1) and *Tropheryma whipplei* (*n* = 1). The most common RNA viruses detected were human immunodeficiency virus (HIV) (*n* = 92), arthropod-borne viruses, also referred to as arboviruses (*n* = 57), and enteroviruses (*n* = 16) (Fig. 2c). Uncommon arboviruses were detected, including St. Louis encephalitis virus^15^, La Crosse virus, Cache Valley virus and Potosi virus, a bunyavirus not previously described in human infections and originally identified by screening of mosquito pools^16^. All 16 enterovirus-positive samples were typeable based on sequence recovered from the VP1 gene, and typing revealed a diversity of genotypes, including D68 and A71 associated with cases of meningoencephalitis and/or acute flaccid myelitis^17–19^. Fungal pathogens detected by mNGS testing consisted of *Coccidioides sp*., including *Coccidioides immitis* (*n* = 14) and *Coccidioides posadasii* (*n* = 2), *Cryptococcus sp*., including *Cryptococcus neoformans* (*n* = 12) and *Cryptococcus gattii* (*n* = 1), *Histoplasma capsulatum* (*n* = 4) and *Fusarium sp*. (*n* = 3). Interestingly, the *Cryptococcus gattii* case was negative by both CSF and serum cryptococcal antigen testing, which has previously resulted in delayed diagnosis and treatment for patients infected by this pathogen^20^. The assay was also able to detect parasitic infections from *Toxoplasma gondii* (*n* = 10), *Balamuthia mandrillaris* (*n* = 3), *Angiostrongylus cantonensis* (*n* = 2) and *Naegleria fowleri* (*n* = 1).

### UCSF patient cohort

Results from mNGS testing of 1,164 samples performed from June 2016 to June 2023 for 1,053 UCSF patients were analyzed and retrospective chart review was performed. Among the 1,053 UCSF patients, 579 were male (55.0%), 160 (15.2%) were children, 893 (84.8%) were adults and 377 (35.8%) were immunocompromised. Each sample represented a single hospitalization or outpatient clinic visit and was thus designated as a separate case, as some patients had multiple samples tested by mNGS. Among the 1,164 cases analyzed by mNGS testing, 1,021 (87.7%) were hospitalized with a median length of stay of 12 days (interquartile range (IQR) 5 − 25 days), 450 (38.7%) were admitted to an intensive care unit (ICU), and 119 (10.2%) died within 60 days of admission. The UCSF cohort consisted of cases of microbiologically- confirmed CNS infection (n = 209, 18%), autoimmune disease or another noninfectious condition (n = 432, 37.1%), prion disease (n = 1, 0.1%), or unknown etiology after 6 months of longitudinal follow-up (n = 522, 44.8%) **(**Table 2**)**. For each case, the average number of microbiological tests performed was 20.2 in total, 6 from CSF and 14.2 from other sample types, and consisted of a mix of culture-based (n = 5.7), nucleic acid amplification (n = 5), antigen (n = 2.7), and serologic (n = 6.8) testing. Among 209 confirmed CNS infectious etiologies, seven cases were polymicrobial, with each causative organism analyzed as a distinct infection for a total of 220 CNS infections. The mNGS positivity rate was higher for immunocompromised (16.7%) than immunocompetent (7.1%) patients (*P* < 0.0001), and higher for meningitis (15.4%, *P* = 0.0466) and meningoencephalitis (17.8%, *P* = 0.0103) than encephalitis (9.9%) patients (Table 2).

**Table 2:** Patient and mNGS test characteristics associated with 1,164 samples from 1,053 patients in UCSF cohort

Patient or mNGS test characteristic		Number (%) of patients (*n* = 1,053 patients)	Number (%) of cases or samples (*n* = 1,164 cases or samples)	Number (%) of infections (*n* = 220 infections)	Number (%) of patients or infections assigned to a specific category with positive mNGS testing	Median length of stay in days [IQR] (*n* = 1,164 cases or samples)
Sex						
	Male	579 (55.0%)				
	Female	474 (45.0%)				
Age						
	<18	160 (15.2%)				
	18–65	630 (59.8%)				
	>65	263 (25.0%)				
Immunosuppression						
	No immunosuppression	676 (64.2%)			48 (7.1%)^a^	
	Any immunosuppression	377 (35.8%)			63 (16.7%)^a^	
	HIV	48 (4.6%)			20 (41.7%)	
	Solid organ transplant	56 (5.3%)			12 (21.4%)	
	Hematopoietic stem cell transplant	45 (4.3%)			8 (17.8%)	
	Chemotherapy	40 (3.8%)			4 (10.0%)	
	Immunomodulatory agents	55 (8.1%)			4 (4.7%)	
	Primary immunodeficiency	16 (1.5%)			1 (6.3%)	
	Other immunosuppression	87 (8.3%)			14 (16.1%)	
Primary clinical syndrome						
	Encephalitis		293 (25.2%)		29 (9.9%)^b^	
	Meningitis		293 (25.2%)		45 (15.4%)^b^	
	Meningoencephalitis		202 (17.4%)		36 (17.8%)^b^	
	Myelitis		69 (5.9%)		1 (1.4%)	
	Other		307 (26.3%)		16 (5.2%)	
Clinical setting and outcomes						
	Inpatient		1,021 (87.7%)			
	ICU		450 (38.7%)			
	Length of stay					12 [5, 25]
	Death at 60 days		119 (10.2%)			
mNGS results^c^						
	Samples with at least one positive result		180 (15.5%)			
	Total number of organisms detected^d^		227			
	Organisms considered incidental (excluded)		85			
	Organisms considered of unclear importance (excluded)		3			
	Organisms considered as positive detection		139			
	Negative detection (no organism detected)		907 (77.9%)			
	Negative detection (single organism, possible contaminant)		27 (2.3%)			
	Negative detection (multiple bacterial/fungal taxa, probable contaminant)		65 (5.6%)			
Final adjudicated diagnosis^e^						
	Noninfectious		432 (37.1%)			
	Prion		1 (0.1%)			
	Unknown		522 (44.8%)			
	Infectious		209 (18.0%)			
Infectious diagnosis category^f^						
	Bacterial			55 (25%)	26 (47.3%)	
	DNA virus			71 (32.3%)	53 (74.6%)	
	RNA virus			30 (13.6%)	21 (70.0%)	
	Fungal			46 (20.9%)	23 (50.0%)	
	Parasitic			18 (8.1%)	12 (66.7%)	

^a^The difference in mNGS positivity rate between no immunosuppression and any immunosuppression is statistically significant (*P* < 0.0001) using the two-tailed chi-squared test without adjustment for multiple comparisons.

^b^The difference in mNGS positivity rate between encephalitis and meningitis (*P* = 0.0466) or meningoencephalitis (*P* = 0.0103) is statistically significant using the two-tailed chi-squared test without adjustment for multiple comparisons.

^c^Samples can fit in more than one category (for example, detection of multiple bacterial/fungal taxa suggesting contamination and positive detection of relevant RNA virus). Samples with multiple taxa detected for bacteria/fungi or single detected taxa corresponding to a commensal and/or environmental microorganism and reported with a comment about potential contamination were considered negative, as they represent likely contamination unless clearly related to the final adjudicated diagnosis. See Methods for a description of the result classification.

^d^This number includes three organisms initially reported as possible contamination and reclassified as positive detection given the clinical context.

^e^Clinical adjudication was performed to determine the etiology of the clinical syndrome by chart review. For the syndrome to be considered infectious, at least one microbiological test had to be positive, and consistent with the clinical adjudication.

^f^Final diagnoses included more than one causative organism for seven samples and were analyzed as separate infections.

**Fig. 3:**
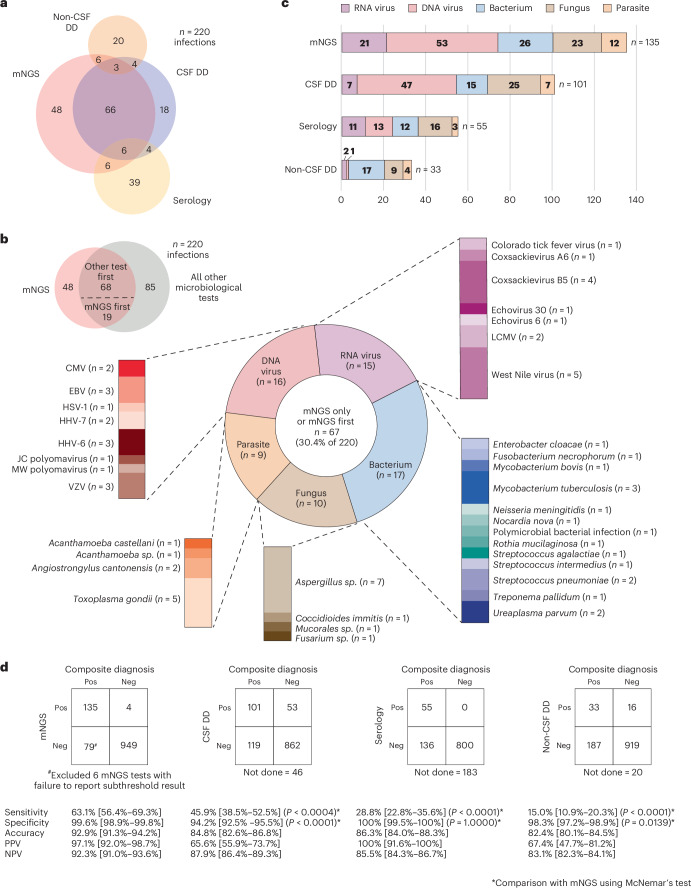
Evaluation of true positive mNGS test results and comparison to other microbiologic tests. **a**, The proportional Venn diagram displays the overlap among four modalities (mNGS, CSF direct detection, non-CSF direct detection and serologic testing) in diagnosis of CNS infections. **b**, Pathogens detected by mNGS testing only (n = 48) or mNGS testing first (*n* = 19). Among the 19 pathogens that were detected by mNGS testing first, 11 pathogens were detected by another microbiologic test run in parallel, whereas 8 were detected by another test, but only for orthogonal confirmation of the initial mNGS positive result. **c**, Number and types of pathogens detected by each of the 4 diagnostic modalities. **d**, 2 × 2 contingency tables showing the comparative performance of mNGS testing compared to other diagnostic modalities. The *P* value is based on comparison between mNGS and a conventional testing modality using the two-sided McNemar’s test. CMV, cytomegalovirus; EBV, Epstein-Barr virus; HHV-6, human herpesvirus 6; HHV-7, human herpesvirus 7; LCMV, lymphocytic choriomeningitis virus; Neg, negative; NPV, negative predictive value; Pos, positive; PPV, positive predective value; VZV, varicella-zoster virus. Source data

We established a composite diagnosis for each case that incorporated all microbiological testing results and clinical adjudication performed independently by three infectious disease physicians, with discrepancies resolved by consensus (Supplementary Dataset 2). Among the 1,164 cases, 180 (15.5%) were positive for the identification of one or more microorganisms by mNGS testing (Table 2 and Methods). Out of the 180 positive cases, 227 organisms were detected, of which 3 had been reported as contaminants but were reclassified as clinically meaningful detections after adjudication (Supplementary Tables 2 and 3). Among the 227 detected organisms, 135 were adjudicated as true positive, 4 as false positive, 85 as incidental detections, such as detection of human immunodeficiency virus 1 (HIV-1) in an infected patient, and 3 as detections of unclear importance for which causality could not be determined (Fig. 3 and Supplementary Table 5). 35 (15.4%) of 227 detected organisms were subthreshold detections, of which 32 (91%) were true positive infections, and the remaining 3 (8.6%) incidental or of unclear importance.

A proportional Venn diagram revealed that among the 135 true positive infections diagnosed by mNGS testing, 48 were made only by this modality, comprising 21.8% of all 220 infectious diagnoses in the UCSF cohort (Fig. 3a,b). An additional 19 infectious diagnoses were made first by mNGS testing, of which 8 were subsequently confirmed by orthogonal testing. mNGS testing was the most sensitive modality for detection of all pathogen types except fungi, which were identified more often by other CSF direct detection (CSF-DD) methods (Fig. 3c). Among 10 fungal infections missed by mNGS testing, including 7 cases of *Cryptococcus neoformans* and 3 of *Coccidioides sp*., 7 were identified by an antigen testing, 2 by culture, and 1 by both antigen testing and culture. Overall, mNGS testing exhibited a sensitivity of 63.1%, specificity of 99.6%, accuracy of 92.9%, positive predictive value of 97.1% and negative predictive value of 92.3% for diagnosis of CNS infections (Fig. 3d). The sensitivity or diagnostic yield of mNGS testing (63.1%) was higher than that for all other modalities, including CSF-DD testing (45.9%, *P* < 0.0004), non-CSF direct detection testing (15.0%, *P* < 0.0001), and indirect serologic testing from serum, plasma, or CSF (28.8%, *P* < 0.0001) (Fig. 3d). When considering only cases diagnosed by CSF-DD testing, the sensitivity of mNGS testing increased to 86% (Supplementary Dataset 2). Sensitivity of mNGS testing was 72.1% for encephalitis, 62.7% for meningitis, 59.4% for meningoencephalitis, 33.3% for myelitis and 62.1% for other syndrome (*P* = 0.5519) (Supplementary Dataset 2).

Turnaround times for the 135 positive mNGS tests from sample collection to result were compared (Supplementary Table 6). The median turnaround time was 9 days (IQR 7–11 days), which was longer than other diagnostic modalities, including CSF-DD testing (4 days (IQR 1–8 days), *P* < 0.0001), non-CSF direct detection testing (6 days (IQR 5–12 days), *P* = 0.0427), and serology (6 days (IQR 3–7.5 days), *P* < 0.0001). Of note, mNGS was generally ordered later than conventional tests, as the laboratory turnaround time was only 3.6 days (Table 1). Thus, we also evaluated the positivity rate based on the time from sample collection to start of processing (Supplementary Table 7). Among all 1,164 mNGS tests, 37.3% were early (<3 days from CSF collection to the start of sample processing), 47.7% were second-line (3–7 days), and 15% were late (>7 days). The positivity rate for mNGS testing was higher for early (12.4%, *P* = 0.0809) and second-line (10.8%, *P* = 0.1564) than for late (7%) testing, although these differences were not statistically significant.

**Fig. 4:**
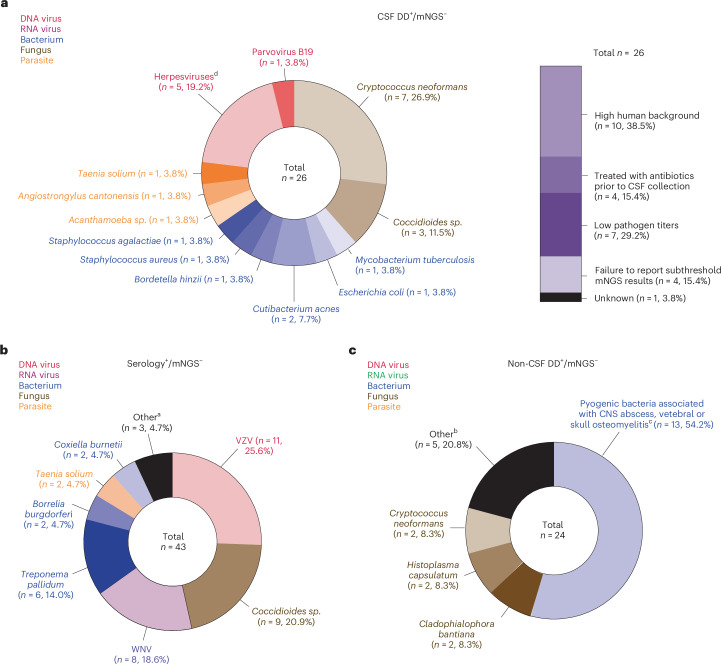
Evaluation of false-negative mNGS test results. **a**, 26 samples were positive by direct detection CSF testing and negative by mNGS testing. Of these false-negative results, 10 (38.5%) were attributed to high DNA host background, a known limitation of mNGS approaches, and 4 (15.4%) to persistent antigen positivity from fungal infection after onset of treatment. **b**, 43 samples were positive by serology and negative by mNGS testing. Most of these false-negative results can be explained by the presumed absence of nucleic acid in the samples at the time of CSF collection, a known limitation of direct detection methods. **c**, 24 samples were positive by non-CSF direct detection testing and negative by mNGS testing. In these cases, the causative pathogen is presumed absent in CSF and only detectable from infected tissue or abscesses. ^a^HSV-1, HSV-2, CMV, EBV and VZV. ^b^*Listeria monocytogenes*, *Sporothrix shenckii*., and EBV. ^c^*Rhizopus sp*., *Candida albicans*, *Balamuthia mandrillaris*, enterovirus and VZV. ^d^*Mycobacterium chelonae*, *Pseudomonas aeruginosa*, *Staphylococcus aureus* (*n* = 3), *Enterococcus faecalis*, *Citrobacteri koseri*, *Streptococcus mitis*, *Cutibacterium acnes*, *Aggregatibacter sp*., *Bacillus cereus*, *Parvimonas micra* and *Streptococcus intermedius*. CMV, cytomegalovirus; CSF DD, CSF direct detection testing; EBV, Epstein-Barr virus; HSV, herpes simplex virus; non-CSF DD, direct detection testing of samples other than CSF; VZV, varicella-zoster virus; WNV, West Nile virus. Source data

We analyzed discrepant results between mNGS testing and other CSF-DD methods. mNGS testing identified 60 infections that were not detected by CSF-DD methods (Supplementary Table 8). Of the 60 mNGS^+^/CSF^−^DD^−^ infections, 19 (31.7%) pathogens were not detected by CSF-DD methods because the diagnosis had not been considered by the treating clinicians *a priori* despite the availability of targeted testing, 11 (18.3%) because a test to detect the pathogen was not readily available (for example, detection of lymphocytic choriomeningitis virus (LCMV) and *Angiostrongylus cantonensis*), and 30 (50%) because conventional testing results were false negative, including 12 (20%) cases of culture-negative mycobacterial or fungal infection and 9 (15%) culture-negative cases attributed to effective antimicrobial treatment prior to CSF collection. Conversely, CSF-DD methods were able to detect 26 infections missed by mNGS testing (Supplementary Table 9). Of the 26 mNGS^−^/CSF DD^+^ infections, 10 (38.5%) were associated with high host background and hence reduced assay sensitivity^14,21^, 4 (15.4%) were in patients with fungal infections who had received prior treatment, 7 (29.2%) were low-titer samples, and 4 (15.4%) were samples with positive subthreshold mNGS test results that had not been reported (Fig. 4a). No explanation for discrepant results was found in 1 (3.8%) mNGS false-negative infection. Notably, all 4 patients with fungal infections missed by mNGS had remained antigen positive for *Cryptococcus sp*. or *Coccidioides sp*. despite a median 12 days of antifungal treatment. Persistence of cryptococcal antigen in CSF weeks to months after effective antifungal therapy has been previously reported^22^.

False-negative mNGS cases with positive CSF or blood serology occurred for 43 infections (Fig. 4b), including varicella-zoster virus (*n* = 11, 25.6%), *Coccidioides sp*. (*n* = 9, 20.9%), and West Nile virus (*n* = 8, 18.6%). These false-negative results can be explained by absence of nucleic acid in CSF at time of collection, a known limitation for all direct detection methods, including mNGS. False-negative mNGS cases with positive non-CSF direct detection testing occurred for 24 infections (Fig. 4c). Many infections were caused by pyogenic bacteria associated with CNS abscesses or skull/vertebral osteomyelitis (*n* = 13, 54.2%), for which the pathogen was only detectable from infected brain tissue or abscess fluid.

## Discussion

In this study, we report analytical results from 7 years of mNGS testing since its inception in 2016. We found that mNGS testing was the most sensitive test for the detection of pathogens in a cohort of patients with clinically severe and diagnostically challenging CNS infections and the only or first test to make the diagnosis in 67 (30.4%) of 220 infections. The test was able to identify a broad array of pathogens that are difficult to detect using conventional methods, including novel, emerging, and/or unexpected microorganisms that were not considered and tested a priori. The higher positivity rate for UCSF compared to non-UCSF patients (16.2% versus 13.9%, *P* = 0.0547) underscores the importance of incorporating additional clinical information from chart review and feedback from treating physicians when interpreting mNGS test results. However, although adding clinical context likely improved the accuracy of mNGS diagnoses for UCSF patients, this information is often not available to reference laboratories performing NGS testing for outside hospitals, as was the case for non-UCSF patients. In addition, local guidelines for ordering mNGS testing at UCSF, including CSF pleocytosis, abnormal imaging, immunocompromised status, and/or *a priori* clinical suspicion of infection based on infectious diseases or neurology consultation, were likely more stringent for UCSF than non-UCSF patients. Importantly, high initial suspicion for infection was found to be an important predictive factor for higher diagnostic yield in another clinical metagenomic study^23^.

This study also indicates that it may be useful to decrease the pre-established reporting thresholds for pathogens associated with low-titer infections, including *Mycobacterium tuberculosis* and *Coccidioides sp*. Our thresholds were initially set by receiver-operator curve analysis using a set of positive and negative control samples during analytic validation^14^. The analysis protocol was subsequently modified to allow subthreshold results to be reported at the discretion of the laboratory director. Over time, reporting thresholds may be permanently lowered for certain pathogens such as *Mycobacterium tuberculosis* that typically cause paucibacillary infections, provided that there are enough cases to validate the new cutoffs.

The overall sensitivity of 63.1% for mNGS testing (86% in cases diagnosed by CSF direct detection methods, including mNGS) is not sufficient to replace conventional microbiologic testing, although the specificity of 99.6% is high. Most of the false-negative mNGS test results are attributed to cases for which the causative pathogen is absent in CSF because of either compartmentalized infection in brain tissue or CNS abscesses^24^ or ‘hit-and-run’ exposures resulting in a very brief period of CNS viremia, such as from West Nile virus^25^. For these mNGS-negative cases, methods based on detecting pathogens from non-CSF sample types and/or indirect detection, such as serology, remain useful. Host transcriptome analysis from RNA sequencing can be a promising approach to further enhance diagnosis^7^. Host transcriptome analysis leverages human gene expression from RNA sequencing data and the development of machine learning based classification models to identify different infections based on the patient host response, including bacterial sepsis^26^, Lyme disease^27^, and tuberculous meningitis^28^.

The results of mNGS testing over 7 years point to at least four potential clinical indications or ‘use cases’ for the assay, (i) detecting unculturable, difficult-to-diagnose organisms, (ii) broadly diagnosing viral infections, (iii) identifying rare, unexpected infections and (iv) aiding in public health investigations of outbreaks. First, many fastidious bacterial species identified by mNGS testing require specialized incubation conditions or routinely fail to grow in culture, including *Bartonella henselae*, *Tropheryma whipplei, Borrelia burgdorferi*, and *Leptospira borgpetersenii*. The mNGS test also detected mycobacteria and fungi that can take weeks to months to grow in culture, including *Histoplasma capsulatum*, *Mycobacterium tuberculosis, Aspergillus sp*., *Rhizopus sp*. and *Coccidioides sp*. These infections not only are difficult to diagnose but often require a large volume of CSF for culture and/or serologic testing that may not be readily available^29^. Importantly, mNGS testing enabled not only detection but also subtyping of emerging fungal pathogens of relevance to public health such as *Cryptococcus gattii*^30^ and *Fusarium solani*^31^.

Second, mNGS testing can be useful for broad-based diagnosis of viral infections. Currently, most clinical microbiology laboratories do not perform viral culture for diagnostic purposes, as detection of viruses has been largely replaced by targeted nucleic acid amplification testing (NAAT) assays^32^. However, NAAT assays in clinical use can only detect one or a few viral pathogen(s). In our study, mNGS testing was able to detect several cases of viral infection that remained undiagnosed because of (i) negative serology and/or PCR testing, (ii) failure to consider a specific viral diagnosis *a priori*, and/or (iii) lack of available reference laboratory testing. mNGS testing was particularly useful for neuroinvasive arboviral infections from flaviviruses (West Nile and Powassan viruses), bunyaviruses (St. Louis encephalitis, Cache Valley, and Potosi viruses), and reoviruses (Colorado Tick Fever virus). Serology, which is commonly used for diagnosis of arboviral infections^33^, can be limited by low specificity, long test turnaround times, limited availability, and potential false-negative results due to a patient’s immunocompromised status and/or delay in mounting a detectable antibody response^34,35^. Notably, although direct detection assays can be less sensitive given the transient period of detectable virus circulating in CSF, mNGS testing was still able to detect many arboviral infections, even those testing negative by serology. Additionally, unlike NAAT testing, mNGS testing enables genotyping of clinically relevant viral subtypes, such as enterovirus A71 and D68 in cases of meningoencephalitis and/or acute flaccid myelitis^17–19^.

Third, mNGS testing can identify rare and/or unexpected infections in patients with undiagnosed CNS infection. For many of these unusual cases, the clinical presentation and history are atypical, and thus the causative pathogen was not considered and tested for by the treating clinicians a priori. Examples include life-threatening and mostly fatal amebic infections from *Balamuthia mandrillaris, Acanthamoeba castellanii* and *Naegleria fowleri*. The availability of diagnostic tests is limited for these parasites, and microscopy or NAAT testing on CSF is often negative, necessitating a brain biopsy to establish the diagnosis if mNGS testing is not available^36^. Two additional examples of unexpected pathogens include cases of *Yersinia pestis* infection in a patient with unexplained neutrophilic meningitis from New Mexico and recurrent coxsackievirus B5 infection in a patient with idiopathic transverse myelitis who was immunocompromised because of rituximab treatment. Other rare and/or unexpected pathogens detected by mNGS testing but missed by conventional microbiologic testing include *Candida albicans, Toxoplasma gondii*, *Taenia solium* (neurocysticerosis), LCMV and *Trypanosoma cruzi*. Of note, targeted diagnostic tests for all these microorganisms are available in specialized reference labs but were not ordered due to lack of clinical suspicion *a priori*.

Fourth, clinical mNGS results can aid in outbreak investigations for detection of pathogens of public health importance. For example, we reported infection from the vaccine strain of yellow fever virus in CSF from a transplant recipient with encephalitis^37^. This case prompted an investigation by the US Centers for Disease Control and Prevention (CDC) of a nationwide transplant-associated outbreak resulting from transfusion-transmission of live yellow fever vaccine virus to a solid organ donor^37^. We also identified the first patient in the United States with *Fusarium solani* infection associated with an outbreak of fungal meningitis in patients undergoing surgical procedures performed under epidural anesthesia in Matamoros, Mexico^31^. This detection triggered a CDC notification recommending testing for all potentially exposed patients in the outbreak^38^.

This study differs substantially from the prospective PDAID study carried out in 2016 to evaluate the performance and clinical impact of the UCSF CSF mNGS test^6^. In contrast to the PDAID study, which prospectively enrolled patients meeting specific inclusion criteria, the current study evaluates the longitudinal performance of mNGS testing over 7 years performed on samples without specific inclusion criteria, from across the United States, and often lacking associated clinical metadata. Furthermore, after publication of the PDAID study^6^, we began to report subthreshold results, as the previously established thresholds^14^ were too conservative for high-consequence pathogens that can be present in CSF at low titers, including *Mycobacterium tuberculosis* and *Coccidioides sp*. Nevertheless, the sensitivity and specificity of mNGS testing compared to other CSF direct detection testing methods are similar between the two studies (86% and 99.6%, respectively, for this study and 80.7% and 98.2%, respectively for the PDAID study).

There are several limitations to our study. The diagnostic workup varied widely from patient to patient, making it impossible to make a true head-to-head comparison of mNGS versus other microbiological assay testing. Bias may also have been introduced by incorporating clinical adjudication as part of the composite diagnosis; however, potential bias was minimized by incorporating independent assessments from 3 different infectious disease physicians. Finally, this study did not formally address clinical indications, actionable impact, and cost-benefit considerations for mNGS, and these analyses are in progress.

Currently, mNGS testing of CSF has limited availability and is often performed as a test of ‘last resort’^39^, as its role in the diagnostic algorithm for CNS infections is yet undefined. The lack of guidelines for the use of mNGS testing has led to its slower adoption by providers, resulting in their hesitance to order testing until far along the clinical course and reducing access to patients. Ultimately, delayed mNGS testing narrows the window of clinical actionability and can decrease diagnostic yield as pathogen titers decline over time, especially in patients treated with empiric antimicrobial therapy. Indeed, our findings show that the diagnostic yield was higher in samples tested within 7 days of CSF collection (Supplementary Table 7), although the differences in yield were not statistically significant. Further investigation is needed to confirm the potential benefit of earlier mNGS testing, which must be weighed against other factors such as turnaround time, cost, availability, and the patient population to be tested (for example, ICU or immunocompromised patients). A reasonable strategy might be to first perform tests that can be completed within a few hours, including multiplex PCR panels such as the BioFire FilmArray Meningitis/Encephalitis panel^40^, herpesvirus PCR and antigen tests for *Cryptococcus sp*., followed by mNGS if the initial round of testing is negative and infection remains part of the differential diagnosis.

The cost of UCSF CSF mNGS testing (~$3,000 per sample as of 2024) is higher than that of conventional microbiological tests and is also prohibitive outside of high-income countries. Costs can potentially be reduced through automation and increasing economies of scale. However, when assessing the value of mNGS, the clinical context for its use should be taken into consideration. This test has historically been used in patients with complex presentations of suspected infectious meningitis, encephalitis, and/or myelitis who are hospitalized with lengths of stay ranging from 3 to 13 days^41^ and an average of 27 microbiological tests ordered per patient^42^. Of note, the patients in our study had 20.2 tests performed on average. In this severely ill patient population, mNGS testing has the potential to reduce overall costs by decreasing the number of tests needed and time to diagnosis, which can decrease lengths of stay and need for invasive diagnostic procedures. Taken together, our results indicate that mNGS testing should be viewed as a complementary yet essential part of the workup for patients with diagnostically challenging CNS infections. Further studies on the clinical impact and cost-effectiveness of mNGS testing will help to define the indications and optimal timing for the assay.

## Methods

### Inclusion and ethics

Retrospective patient chart review and analysis of patient clinical CSF results were performed using a biobanking protocol (#10-01990) with waiver of consent approved by the UCSF Institutional Review Board for UCSF and non-UCSF patients. All UCSF patients undergoing CSF mNGS testing from June 2016 to June 2023 were included regardless of their age, gender, race, or ethnicity. No sex or gender analyses were performed, nor was the data reported as disaggregated for sex and gender, as these factors were not considered relevant in the evaluation of a diagnostic test for CNS infections intended to be broadly applicable for patients, irrespective of sex and gender.

### CSF mNGS Testing

The UCSF CSF mNGS test was developed to facilitate the broad identification of pathogens in diagnostically challenging CNS infections. Our previously reported clinically validated assay workflow consisted of (i) nucleic acid extraction, (ii) microbial enrichment using antibody-based removal of methylated host DNA for DNA libraries and DNase treatment for RNA libraries, (iii) library preparation and pooling in equimolar concentrations, and (iv) sequencing on an Illumina NextSeq 550 or NextSeq 550Dx instrument targeting 5–20 million reads per library^14^. A complete list of reagents, supplies, and devices used is provided in Supplementary Table 10. Raw reads were analyzed using SURPI+, a bioinformatics analysis pipeline for pathogen identification that was modified for clinical use^14,43^. To provide results visualization and assist in laboratory director review and reporting, automated results from SURPI+ included heat maps of raw and normalized read counts per million (RPM) for each sample and a results summary in an Excel spreadsheet format. A laboratory director (CYC or SM) reviewed and interpreted all mNGS test results before reporting the findings.

QC metrics for the assay included a minimum of 5 million preprocessed reads per sample, >75% of data with quality score >30, and successful detection of all 7 representative organisms in the positive control and the internal spiked T1 and MS2 phage controls. High host background was identified by the detection of <100 RPM for the internal spiked T1 and MS2 controls in DNA and RNA libraries, respectively^14,21^. A low number of preprocessed reads for a library was also identified if below a pre-established cutoff of 5 million reads. For high host background and/or a low number of preprocessed reads, negative mNGS results were reported with a comment regarding potential decreased sensitivity of mNGS testing. A threshold criterion of ≥3 nonoverlapping viral reads aligning to the target viral genome at the genus and/or species level was considered a positive detection for virus identification, whereas an RPM ratio threshold of 10 was considered positive for bacteria, fungi and parasite detection^14^. Following the completion of a prospective study to evaluate clinical utility and diagnostic yield^6^, the reporting algorithm was modified to allow additional comments regarding subthreshold detections of high-consequence organisms such as *Mycobacterium tuberculosis* and *Coccidioides immitis* at the discretion of the laboratory director. A subthreshold detection was defined as detection of reads aligning to a microorganism at levels below the pre-established cutoffs of ≥3 for viruses or RPM ratio of 10 for bacteria, fungi, and parasites.

Contaminant organisms corresponding to potential pathogens in the external positive and negative control samples were recorded longitudinally in a contaminant database for over a 3-month window and available to the laboratory director when results are reviewed and reported (Supplementary Dataset 3). All reads in a sample corresponding to a contaminant organism were automatically flagged for review by the laboratory director. Organisms that are detected above the pre-established positive thresholds in clinical samples can be reported as contamination at the discretion of the laboratory director based on several factors: (i) if the organisms were part of the contaminant database at the time of review, (ii) if the sample contained multiple organisms known to be commensal or environmental species, and/or (iii) if there was evidence of cross-contamination from a high-titer organism in another sample on the current run. When reviewing the sequencing results for this study, organisms were classified as contaminants when a comment regarding possible or likely contamination was added by the laboratory director at the time of reporting.

### All patient cohort

mNGS test results analyzed in this study included those from all samples received from June 2016 to April 2023 (*n* = 4,828). Demographic information that was available for all patients included age, sex and geographic location by state. Clinical information was available for a subset of cases, including UCSF and non-UCSF patients for whom detection of an unusual microorganism prompted a clinical microbial sequencing board (CMSB) discussion with the treating provider, and UCSF patients for whom information was available from the electronic medical records. For each sample, run QC metrics and sequencing results were evaluated, along with overall turnaround time from sample collection to result and laboratory turnaround time from start of processing to result.

### UCSF patient cohort

The UCSF cohort consisted of 1,164 samples or cases from 1,053 patients collected from June 2016 to June 2023 and was assessed by retrospective chart review to determine the performance and utility of mNGS testing in diagnosing CNS infections compared to other assays. All requests for mNGS testing were reviewed by the laboratory director, and testing was approved if patients met one of more of the following criteria: (i) CSF pleocytosis (≥5 white blood cells/mm^3^), (ii) brain biopsy that suggested a likely infectious etiology, (iii) immunocompromised patient with strong suspicion of infection based on clinical presentation, laboratory testing, and/or radiographic imaging and (iv) recommendation for mNGS testing after neurology and/or infectious diseases consultation. A final composite diagnosis for the UCSF cohort was established through clinical adjudication and review of all clinical microbiologic tests that were performed for the patient, including mNGS testing (Supplementary Dataset 2).

### Comparative performance of mNGS testing

Clinical adjudication and chart review were performed independently by three infectious disease physicians (P.B., M.L.T. and C.Y.C.), who categorized each case as bacterial, viral, fungal, parasitic, noninfectious (for example, autoimmune disease), prion-associated, or unknown. Any discrepancies in the assigned final diagnosis were resolved by mutual agreement after re-review and discussion. A case was considered to have an infectious diagnosis if at least one test result, whether by mNGS or conventional microbiological testing, was positive. If one case included more than one causative pathogen, each organism was considered individually for performance assessment. All clinical microbiological tests relevant to the final diagnosis were recorded for each patient and assigned to one of four different categories: (1) mNGS testing; (2) direct detection testing from CSF (culture, antigen detection and NAAT); (3) direct detection testing from sample types other than CSF, such as brain biopsy tissue and/or plasma (culture, antigen detection, and NAAT); and (4) indirect serologic testing from CSF or blood. For each positive test, the turnaround time from sample collection to test result was also recorded. mNGS test results were further classified as (i) positive, (ii) detection of a single organism corresponding to a commensal and/or environmental species that was reported as possible contamination (Supplementary Table 2), (iii) detection of multiple bacterial/fungal taxa that was reported likely contamination (Supplementary Table 3), (iv) detection of organisms considered incidental and thus not involved in the pathogenesis of the CNS infection, or (v) detection of organisms of unclear importance where the causality of the pathogen could not be determined (Supplementary Table 4). For the performance assessment, both possible (single taxon) and probable (multiple taxa) contaminants were considered as negative, unless clearly related to the final adjudicated diagnosis, whereas detection of organisms considered incidental or of unclear importance were excluded from the analysis. Each pathogen was considered individually in cases of co-infection. A true positive was defined as positive mNGS detection of a presumed pathogenic organism that was the cause of the patient’s CNS infection, as determined by clinical adjudication and chart review. A false positive was defined as positive mNGS detection of a presumed pathogenic organism that was not the cause of the patient’s infection. In some instances, two categories were assigned to a single result (for example, a false-positive result on mNGS testing that also missed the true diagnosis was assigned a 1 for the false-positive and false-negative cells). If no test corresponding to the diagnostic modality was performed for a sample (for example, no serology performed for a patient with an infection), then the result was excluded from performance analysis for that category. Six samples were also excluded from the performance analysis because reads to the causative organisms were present at subthreshold levels, below the pre-established cutoffs, but had not been reported (Supplementary Tables 9 and 11).

### Statistical analyses

Statistical analyses were performed using the Python scipy package (version 1.14) as implemented in Python (version 3.12.4) or GraphPad Prism software (version 10.1.0). The Mann-Whitney test was used to assess differences in turnaround time. The Chi-squared test was used to compare sequencing results between UCSF and non-UCSF samples, mNGS assay sensitivity according to clinical syndrome, and positivity rates according to the timing of mNGS testing. The McNemar test was used to compare sensitivity and specificity differences between mNGS and conventional microbiologic testing. Proportional Venn diagrams were constructed using the DeepVenn web application^44^.

### Reporting summary

Further information on research design is available in the Nature Portfolio Reporting Summary linked to this article.

## Online content

Any methods, additional references, Nature Portfolio reporting summaries, source data, extended data, supplementary information, acknowledgements, peer review information; details of author contributions and competing interests; and statements of data and code availability are available at 10.1038/s41591-024-03275-1.

## Supplementary information

Supplementary Information Supplementary Tables 1–11

Reporting Summary

Supplementary Dataset 1 CSF mNGS results for the 4,828 samples/cases in the study.

Supplementary Dataset 2 Performance of CSF mNGS testing compared to other diagnostic modalities and by syndrome.

Supplementary Dataset 3 Potentially pathogenic microbial contaminants detected in control samples by CSF mNGS testing and recorded in a pathogen contaminant database.

## Source data

Source Data Fig. 1 Statistical Source Data

Source Data Fig. 2 Statistical Source Data

Source Data Fig. 3 Statistical Source Data

Source Data Fig. 4 Statistical Source Data

## Data Availability

CSF mNGS results for the 4,828 samples/cases in the study are available in Supplementary Dataset 1. The performance of CSF mNGS testing compared to other diagnostic modalities and by syndrome is available in Supplementary Dataset 2. Potentially pathogenic microbial contaminants detected in control samples by CSF mNGS testing and recorded in a pathogen contaminant database are available in Supplementary Dataset 3. Metagenomic reads from CSF samples from UCSF patients in this study were depleted of human host sequences and have been submitted to the National Center for Biotechnology Information (NCBI) BioProject database under accession number PRJNA1143941 and umbrella accession number PRJNA234047. Please contact the corresponding author (C.Y.C.) regarding data access for non-UCSF patients. Source data are provided with this paper.

## Code availability

The SURPI+ bioinformatics pipeline is described in prior publications^21,22^. The code for SURPI+ includes proprietary algorithms for taxonomic classification, filtering and pathogen software that have been filed under US patent 11380421, ‘Pathogen detection using next-generation sequencing’. Please contact the University of California Office of Technology Management regarding access to and use of the software.
