# Supplementary material for: Seven-year performance of a clinical metagenomic next-generation sequencing test for diagnosis of central nervous system infections
Source: Nat Med. 2024 Nov 12;30(12):3522–33. doi: 10.1038/s41591-024-03275-1 (PMC11645279; doi:10.1038/s41591-024-03275-1)
Supplement: Supplementary file 1 — Supplementary Tables 1–11 [file 41591_2024_3275_MOESM1_ESM.pdf]

nature medicine

Supplementary information https://doi.org/10.1038/s41591-024-03275-1

Seven-year performance of a clinical metagenomic next-generation sequencing test for diagnosis of central nervous system infections

In the format provided by the authors and unedited

Supplementary Table 1. List of bacteria species or genera detected by CSF mNGS testing.

**Non-fastidious pathogens**	**N**	**Uncommon and/or difficult to diagnose pathogens**	**N**
Streptococcus sp.	24	*Mycobacterium tuberculosis*	13
Klebsiella sp.	12	*Mycobacterium sp., non-tuberculosis*	7
Staphylococcus sp.	9	*Nocardia farcinica*	3
Enterococcus sp.	8	*Borrelia burgdorferi*	2
Haemophilus sp.	7	*Mycoplasma hominis*	2
Neisseria sp.	4	*Treponema pallidum*	2
Serratia sp.	4	*Ureaplasma parvum*	2
Citrobacter sp.	3	*Actinomyces oris*	1
Cutibacterium sp.	2	*Bartonella henselae*	1
Enterobacter sp.	2	*Borrelia miyamotoi*	1
Pseudomonas sp.	2	*Chlamydia psittaci*	1
Acinetobacter sp.	1	*Cronobacter sakazakii*	1
Bacteroides sp.	1	*Fusobacterium necrophorum*	1
Corynebacterium sp.	1	*Fusobacterium nucleatum*	1
Cronobacter sp.	1	*Gardnerella vaginalis*	1
Leuconostoc sp.	1	*Kingella kingae*	1
Moraxella sp.	1	*Legionella sp.*	1
Pantoea sp.	1	*Leptospira borgpetersenii*	1
Proteus app.	1	*Mycoplasma pneumoniae*	1
Rothia sp.	1	*Tropheryma whipplei*	1
Shewanella sp.	1	*Ureaplasma urealyticum*	1
		Yersinia pestis	1

Supplementary Table 2. CSF mNGS detection of a single organism reported as possible contamination.

mNGS result	Diagnosis category	Consistent with clinical presentation?
Corynebacterium sp.	Bacterial	Yes[a]
Staphylococcus aureus	Bacterial	Yes[b]
Pantoea spp.	Non-infectious	No
Staphylococcus haemolyticus	Non-infectious	No
Stenotrophomonas rhizophila	Bacterial *(Borrelia burgdorferi)*	No
Deinococcus proteolyticus	Non-infectious	No
Lactobacillus reuteri	Non-infectious	No
Balamuthia mandrillaris, Micrococcus sp.	Parasitic *(Balamuthia mandrillaris)*	No
Candida tropicalis	Non-infectious	No
Pseudomonas sp.	Non-infectious	No
Propionibacterium sp	Unknown	No
Cutibacterium avidum	Unknown	No
Corynebacterium spp	Non-infectious	No
Bacillus infantis	Unknown	No
Sphingomonas sp.	Unknown	No
Staphylococcus warneri	Unknown	No
Clostridium botulinum	Unknown	No
Lactobacillus delbrueckii	Unknown	No
Pantoaea ananatis	DNA virus (VZV)	No
Enterobacter cloacae	Unknown	No
Staphylococcus warneri	Non-infectious	No
Staphylococcus hominis	Non-infectious	No
Shewanella baltica	Unknown	No
Leuconostoc sp.	Non-infectious	No
Pantoea sp.	Unknown	No
Staphylococcus epidermidis	Unknown	No
Cutibacterium avidum	Unknown	No

[a]meningitis in a patient with a ventriculo-peritoneal shunt. CSF culture also positive for *Corynebacterium sp.*
[b]nosocomial meningitis in a patient post neurosurgery. CSF culture also positive for *Staphylococcus aureus.*

Supplementary Table 3. CSF mNGS results with detection of multiple bacterial and/or fungal taxa (n=65).

mNGS result	Diagnosis category	Consistent with clinical presentation?
Porphyromonas gingivalis, Prevotella dentalis, Streptococcus milleri	Bacterial	Yes[a]
Micrococcus luteus, Pantoea sp.	Unknown	No
Rothia dentocariosa, Streptococcus sanguinis,and Streptococcus parasanguinis	Non-infectious	No
Morganella morganii and others	Unknown	No
Sphingomonas sp, Clavibacter sp	Unknown	No
Bifidobacterium dentiuym, Streptococcus mutans, Methylobacterium populi	Non-infectious	No
Pantoea spp., Corynebacterium ureicelerivorans	Bacterial *(Streptococcus agalactiae)*	No
Prevotella, Porphyoromonas, Streptococcus, Haemophilus spp	Non-infectious	No
Pantoea spp, Bifidobacterium thermophilum	Non-infectious	No
Pseudomonas putida and others	Unknown	No
Bacillus megaterium, Rhodococcus equi	Unknown	No
Corynebacterium spp and others	Unknown	No
Multiple bacterial taxa	Unknown	No
Klebsiella pneumoniae, Enterobacter cloacae, Citrobacter freundii	Unknown	No
Lactobacillus acidophilus and others	Non-infectious	No
Streptococcus agalactiae, Exiguobacterium sp., HIV.1, *Cryptococcus neoformans*	Fungal *(C. neoformans)* and Bacterial (*Streptococcus agalactiae*)	No
Prevotella melanogenica, Streptococcus sp	Non-infectious	No
Multiple bacterial taxa	Unknown	No
Pseudomonas simiae ,Streptococcus salivarius, Bacteroides vulgatus	Non-infectious	No
Geobacillus sp.,Enterobacter sp.,and Leuconostoc sp., Candida famata	Unknown	No
Corynebacterium urealyticum and others	Unknown	No
Lactobacillus, Pantoea, Moraxella catarrhalis	Unknown	No
Enterobacter cloacae, Rothia dentocariosa, HIV	RNA virus (HIV)	No
Chroococcidiopsis thermalis	Unknown	No
Streptococcus lutitiensis, Deinococcus proteolyticus, Lactobacillus animalis	Non-infectious	No
Cutibacterium acnes and others	Non-infectious	No
Multiple bacterial taxa	Unknown	No
Blastococcus saxobsidens, Geodermatophilus obscurus, Bifidobacterium thermophilum, Granulibacter bethesdensis, Modestobacter marinus, CMV	DNA virus (CMV)	No
Micrococcus luteus, Streptococcus sp., Dietzia sp	Non-infectious	No
Megamonas hypermegale, Lactobacillus reuteri, Anoxybacillus flavithermus	Non-infectious	No
Modestobacter marinus, Azospirilllum lipoferum, Spirosoma linguale, Variovarox paradoxus	Non-infectious	No
Staphylococcus aureus, Staphylococcus epidermidis, Micrococcus luteus, Serratia marcescens	Unknown	No
Penicillium rubrens and others	Non-infectious	No
Bacillus licheniformis, Micrococcus luteus, Gordonia bronchialis	Unknown	No
Streptococcus thermophilus, Bifidobacterium animalis, Corynebacterium ureicelerivorans	Non-infectious	No
Micrococcus luteus, Klebsiella pneumoniae, Kytococcus sendentarius	Non-infectious	No
Trichophyton rubrum and others	Unknown	No
Lactobacillus sakei, Klebsiella pneumoniae, Acidovorax sp., Rhodococcus erythropolis	Non-infectious	No
Gardnerella vaginalis, Corynebacterium sp., Staphylococcus auricularis	Non-infectious	No
Staphylococcus pettenkoferi, Mycobacterium paragordonae, Bacillus coagulans, Neisseria sicca, Rothia mucilaginosa	Unknown	No
Mycobacterium choelonae and others	Unknown	No
Bifidobacterium adolescentis and others	Unknown	No
Micrococcus, Pseudomonas, Corynebacterium sp, Cryptococcus neoformans, JC polyomavirus, HIV1	Fungal *(C. neoformans)*	No
Acinetobacter radioresistens, Sphingomonas sp., Rhizobium sp.,Stenotrophomonas sp.	Non-infectious	No
Corynebacterium sp., Staphylococcus sp., Malassezia sp., Anthracocystis sp, Tilletiopsis sp.	Non-infectious	No
Porphyomonas sp.,Corynebacterium sp., Stenotrophomonas sp.	Non-infectious	No
Acinetobacter lwoffii, Rothia mucilaginosa, Staphylococcus sp.	Unknown	No
Staphylococcus sp., Corynebacterium sp., Rothia mucilaginosa	Unknown	No
Streptococcus thermophilus, Gardnerella vaginalis, Lactobacillus plantarum, Cutibacterium acnes	Non-infectious	No
Bacteroides sp., Enterococcus faecium, Corynebacterium sp., Cutibacterium acnes	Non-infectious	No
Enterococcus cecorum, Chryseobacterium haifense, Bacillus flexus	Unknown	No
Neisseria sp,Corynebacterium sp., Haemophilus parainfluenzae	Non-infectious	No
Pseudomonas fluorescens, Prevotella sp., Bacteroides vulgatus	Unknown	No
Acineotbacter sp.,Capnocytophaga sp., Lactobacillus crispatus	Unknown	No
Staphylococcus pettenkoferi,Chryseobacterium haifense, Corynebacterium sp.	Fungal *(Coccidioides sp.)*	No
Corynebacterium striatum, Serratia grimesii	Unknown	No
Staphylococcus cohnii,Chryseobacterium haifense, Gordonia bronchialis	Non-infectious	No
Pseudomonas sp., Capnocytophaga sp., Bifidobacterium thermophilum	Unknown	No
Lactobacillus crispatus, Propionibacterium sp., Staphylococcus aureus	Non-infectious	No
Haemophilus parainfluenzae, Pseudomonas sp., Acinetobacter radioresistens	Unknown	No
Neisseria subflava, Rothia mucilaginosa, and Streptococcus thermophilus, Penicillium rubens, Aspergillus glaucus	Unknown	No
Multiple bacterial taxa, HIV1, Cryptococcus neoformans	Fungal *(C. neoformans)*	No
Bacteroides spp and others	Unknown	No
Enterococcus faecalis, Lactobaillus crispatus, Bifidobacterium dentium, Candida famata	Non-infectious	No
Rhodococcus sp., Enterococcus cecorum, Staphylococcus hominis, Pseudomonas koreensis,Sphingomonas taxi	Non-infectious	No

[a]Odontogenic infection with bilateral sinus cavernous thrombosis with neutrophilic pleocytosis and negative CSF culture.

Supplementary Table 4. CSF mNGS results of unclear significance (n=3).

mNGS result	Diagnosis category	Clinical description
Corynebacterium spp.	Unknown	Meningoencephalitis of unknown etiology with workup negative for neoplasia and autoimmune cause. Patient was treated with vancomycin and eventually improved but diagnosis still unsure.
Mycoplasma hominis	Unknown	Nosocomial meningitis with subthreshold detection of *Mycoplasma hominis*. Patient improved without directed therapy against *Mycoplasma sp.* but role in pathogenesis unclear.
Candida albicans	Unknown	Myelopathy in a patient following intravenous drug use. *Candida sp*. positive by mNGS but not by conventional microbiology tests with uncertain diagnosis.

Supplementary Table 5. False positive mNGS results description (n=4). Abbreviations: autoimmune and non-infectious (AINI), central nervous system (CNS), cerebrospinal fluid (CSF), interquartile range (IQR), metagenomics next-generation sequencing (mNGS), turnaround time (TAT).

mNGS result	Diagnosis category	Action upon result	Compatible with clinical presentation	Clinical description
Varicella zoster virus	Unknown	No	No	Encephalopathy with mild CSF pleocytosis that resolved without antiviral treatment.
Dengue virus, *Candida parapsilosis*	AINI	No	No	Diagnosis of CNS neoplastic metastasis. Target detected not compatible with clinical presentation and epidemiology.
Staphylococcus aureus	AINI	No	No	Diagnosis of diffuse idiopathic arachnoiditis. Target detected not compatible with clinical presentation.
Enterococcus faecalis	Unknown	No	No	Diagnosis of idiopathic myelitis. Target detected not compatible with clinical presentation.

Supplementary Table 6. Comparison of turnaround time between mNGS and other microbiologic test modalities for cases of confirmed infection.

Test modality	Median turnaround time [interquartile range][a]	Range (Min–Max)	*P*-value[b]
mNGS, total time from CSF collection to result	9 days [7–11 days]	4–32 days	
mNGS, laboratory time from start of extraction to result	3.6 days [3.3–4.5 days]	2.3–10.4 days	
Direct detection, CSF samples	4 days [1–8 days]	0–42 days	<0.0001
Serology	6 days [3–7.5 days]	1–49 days	<0.0001
Direct detection, non-CSF samples	6 days [5–12 days]	0 –35 days	0.0427

[a]turnaround time was only considered for the first test in each modality that yielded a result concordant with the final infectious diagnosis

[b]comparison of turnaround times between mNGS and other test modalities by two-sided Mann-Whitney testing

Supplementary Table 7. CSF mNGS positivity rates based on timing of testing.

Timing of CSF mNGS testing[a]	Number of mNGS tests (n=1,129)[b]	Positivity rate[c]
≤3 days after CSF collection (early testing)	421 (37.3%)	52 (12.4%)
3-7 days after CSF collection (second-line testing)	538 (47.7%)	58 (10.8%)
>7 days after CSF collection (late testing)	170 (15%)	12 (7%)

[a]timing is based on the number of days after CSF collection before the start of sample processing in the laboratory, which is inclusive of the time between CSF collection and the clinical decision to order mNGS testing and the additional time incurred due to the requirement for batching samples to fill up a sequencing run

[b]time of CSF collection was missing for 35 out of 1,164 samples

[c]A comparison of the differences in positivity rate yields $P = 0.0877$ when comparing (early or second-line) with late testing; $P = 0.1971$ when comparing early with (second-line or late testing); and $P = 0.0809$ when comparing early with late testing. *P*-values were calculated using the two-tailed Chi-squared test without adjustment for multiple comparisons

Supplementary Table 8. Diagnosis made by mNGS and negative by other direct detection tests on CSF (n=60). Abbreviations: HHV, human herpesvirus; HSV, herpes simplex virus; LCMV, lymphocytic choriomeningitis virus; MW, Malawi polyomavirus.

DNA virus (n=12)	RNA virus (n=14)	Bacteria (n=18)	Fungi (n=8)	Parasite (n=8)
Cytomegalovirus	Colorado tick fever virus	*Enterobacter cloacae*	*Aspergillus sp.* (n=5)	*Ancathamoeba castellani*
Epstein-Barr virus (n=2)	Coxsackievirus A6	*Fusobacterium necrophorum*	*Coccidioides immitis* (n=2)	*Balamuthia mandrillaris* (n=2)
HHV-7 (n=2)	Coxsackievirus B5 (n=3)	*Klebsiella aerogenes*	*Mucorales sp.*	*Toxoplasma gondii* (n=5)
HHV-6 (n=2)	Echovirus 30	*Mycobacterium bovis*		
JC virus	Echovirus 6	*Mycobacterium tuberculosis* (n=2)		
Varicella zoster virus (n=2)	LCMV (n=2)	*Neisseria meningitidis*		
HSV-1	West Nile Virus (n=5)	*Nocardia nova*		
MW polyomavirus		Polymicrobial		
		Streptococcus agalactiae		
		Streptococcus intermedius		
		Streptococcus pneumoniae (n=2)		
		Treponema pallidum (n=2)		
		Tropheryma whipplei		
		Ureaplasma parvum (n=2)		

Supplementary Table 9. False negative CSF mNGS test results versus other direct detection tests (n=26). Abbreviations: Ag, antigen; CMV, cytomegalovirus; CrAg, cryptococcal antigen; Cx, culture; EBV, Epstein-Barr virus; HSV, herpes simplex virus; PCR, polymerase chain reaction; uPCR, universal PCR; VZV, varicella-zoster virus.

Organism	Other Direct Detection Test	Infection Category	Reason for Negative CSF mNGS Testing
Acanthamoeba sp.	PCR	Parasitic	Failure to report subthreshold result
Angiostrongylus cantonensis	PCR	Parasitic	Failure to report subthreshold result
Bordetella hinzii	Cx	Bacterial	High human background
CMV	PCR	DNA virus	low positive sample
Coccidioides sp.	Ag	Fungal	Treated before CSF collection
Coccidioides sp.	Cx	Fungal	High human background
Coccidioides sp.	Ag	Fungal	Treated before CSF collection
Cryptococcus neoformans	Ag	Fungal	low positive sample
Cryptococcus neoformans	Ag	Fungal	Failure to report subthreshold result
Cryptococcus neoformans	Ag	Fungal	High human background
Cryptococcus neoformans	Cx	Fungal	low positive sample (CrAg negative)
Cryptococcus neoformans	Ag	Fungal	Treated before CSF collection
Cryptococcus neoformans	Ag/Cx	Fungal	Treated before CSF collection
Cryptococcus neoformans	Ag/Cx	Fungal	High human background
Cutibacterium acnes	Cx	Bacterial	High human background
Cutibacterium acnes	Cx	Bacterial	Unknown
EBV	PCR	DNA virus	low positive sample
Escherichia coli	Cx	bacterial	High human background
HSV	PCR	DNA virus	High human background
HSV-2	PCR	DNA virus	High human background
Mycobacterium tuberculosis	Cx	Bacterial	High human background
Parvovirus B19	PCR	DNA virus	low positive sample
Staphylococcus aureus	Cx	Bacterial	High human background
Streptococcus agalactiae	uPCR	Bacterial	low positive sample
Taenia sp.	Ag	Parasitic	Failure to report subthreshold result
VZV	PCR	DNA virus	low positive sample

Supplementary Table 10. List of kits, reagents, and instruments used for the UCSF CSF mNGS test. Abbreviations: ATCC, American Tissue Culture Collection; CFU, colony forming units; CMV, cytomegalovirus; NTC, no-template control, UCSF, University of California, San Francisco.

**Reagent or Kit**	**Manufacturer**	**Category**
Escherichia coli bacteriophage T1	ATCC 11303-B1	DNA internal control
Escherichia coli bacteriophage MS2	ATCC 15597-B1	RNA internal control
EZ1 Virus Mini Kit v2.0	Qiagen	Nucleic acid extraction
CMV	Cultured strain at UCSF, 1000 copies/mL	External control spike-in (DNA virus)
HIV-1	Positive donor from American Red Cross, 5000 copies/mL	External control spike-in (RNA virus)
Streptococcus agalactiae	ATCC 13813, 50 CFU/mL	External control spike-in (Gram-positive bacterium)
Klebsiella pneumoniae	ATCC 13883, 50 CFU/mL	External control spike-in (Gram-negative bacterium)
Cryptococcus neoformans	ATCC 66031, 1 CFU/mL	External control spike-in (yeast)
Aspergillus niger	ATCC 16888, 500 CFU/mL	External control spike-in (mold)
Toxoplasma gondii	ATCC 40050, 50 organisms/mL	External control spike-in (parasite)
NTC	Qiagen EZ1 elution buffer	No-template Control
NEB Microbiome Enrichment Kit	New England Biolabs	Enrichment for microbial DNA
Turbo DNase	Thermo Fisher	Enrichment for viral RNA
Baseline DNase	Thermo Fisher	Enrichment for viral RNA
RNA Clean & Concentrator Kit	Zymo Research	Enrichment for viral RNA
Random hexamers	Thermo Fisher	cDNA synthesis
SuperScript III buffer	Thermo Fisher	cDNA synthesis
Sequenase enzyme	Affymetrix	cDNA synthesis
DNA Clean & Concentrator Kit	Zymo Research	Library preparation
Nextera XT DNA Library Prep Kit	Illumina, San Diego, CA	Library preparation
AMPure XP beads	Beckman Coulter	Library preparation
Phusion enzyme	Thermo Fisher	Library preparation
Qubit dsDNA HS Assay Kit	Thermo Fisher	Library preparation
High Sensitivity DNA kit	Agilent	Library preparation
**Instrument**	**Manufacturer**	**Category**
FastPrep-24 bead beater	MP Biomedicals	Sample processing
EZ1 BioRobot	Qiagen	Nucleic acid extraction
Agilent 2100 Bioanalyzer	Agilent	DNA/RNA sequencing library preparation
Illumina HiSeq 1500 or NextSeq 550Dx sequencer	Illumina, San Diego, CA	Library sequencing

Supplementary Table 11. Failure to report subthreshold CSF mNGS test results (n=6).
Abbreviations: CMV, cytomegalovirus.

mNGS Report	Microorganism	Diagnosis Category	Clinical Description
Negative	*Acanthamoeba sp.*	Parasitic	Patient with recurrent glioblastoma admitted with acute severe encephalopathy and seizures with likely infectious meningoencephalitis and multifocal brain lesions. *Acanthamoeba* PCR positive on CSF. Review of mNGS results showed detection of reads to *Acanthamoeba sp*. at a subthreshold level. This result was not reported.
Negative	*Taenia solium*	Parasitic	Patient with neurocysticercosis (diagnosed by serum cysticercus antibody and suggestive imaging) complicated by hydrocephalus requiring VP shunt with complex hospital course. Review of mNGS run showed detection of reads to *Taenia sp.* at a subthreshold level. This result was not reported.
Negative	*Taenia sp.*	Parasitic	Patient with history of subarachnoid neurocysticercosis admitted with progressive neurologic symptoms with spinal involvement and communicating hydrocephalus and mixed CSF pleocytosis on lumbar puncture. *Taenia* antigen was positive in CSF. Review of mNGS results showed detection of reads to *Taenia sp.* at a subthreshold level. This result was not reported.
CMV	*Coccidioides sp.*	Fungal	Patient with hydrocephalus and meningitis serum and CSF serology by complement fixation positive for *Coccidioides* Ab. Review of mNGS results showed detection of reads to *Coccidioides sp.* at a subthreshold level. This result was not reported.
Negative	*Cryptococcus neoformans*	Fungal	Patient with cryptococcal meningitis with positive cryptococcal antigen testing from CSF and serum. Review of mNGS results showed detection of reads to *Cryptococcus sp.* at a subthreshold level. This result was not reported.
Negative	*Angiostrongylus cantonensis*	Parasitic	Patient with eosinophilic meningitis after trip to Tahiti. PCR testing for *Angiostrongylus cantonensis* from CSF was positive. Review of mNGS results showed detection of reads to *Angiostrongylus cantonensis* at a subthreshold level. This result was not reported.
