# Supplementary material for: Seven-year performance of a clinical metagenomic next-generation sequencing test for diagnosis of central nervous system infections
Source: Nat Med. 2024 Nov 12;30(12):3522–33. doi: 10.1038/s41591-024-03275-1 (PMC11645279; doi:10.1038/s41591-024-03275-1)
Supplement: Supplementary file 2 — Reporting Summary [file 41591_2024_3275_MOESM2_ESM.pdf]

nature portfolio

Corresponding author(s): Charles Chiu
Last updated by author(s): Aug 7, 2024

Reporting Summary

Nature Portfolio wishes to improve the reproducibility of the work that we publish. This form provides structure for consistency and transparency in reporting. For further information on Nature Portfolio policies, see our Editorial Policies and the Editorial Policy Checklist.

Statistics

For all statistical analyses, confirm that the following items are present in the figure legend, table legend, main text, or Methods section.

n/a	Confirmed	
☐	☒	The exact sample size (*n*) for each experimental group/condition, given as a discrete number and unit of measurement
☐	☒	A statement on whether measurements were taken from distinct samples or whether the same sample was measured repeatedly
☐	☒	The statistical test(s) used AND whether they are one- or two-sided<br>*Only common tests should be described solely by name; describe more complex techniques in the Methods section.*
☒	☐	A description of all covariates tested
☐	☒	A description of any assumptions or corrections, such as tests of normality and adjustment for multiple comparisons
☐	☒	A full description of the statistical parameters including central tendency (e.g. means) or other basic estimates (e.g. regression coefficient) AND variation (e.g. standard deviation) or associated estimates of uncertainty (e.g. confidence intervals)
☐	☒	For null hypothesis testing, the test statistic (e.g. *F*, *t*, *r*) with confidence intervals, effect sizes, degrees of freedom and *P* value noted<br>*Give P values as exact values whenever suitable.*
☒	☐	For Bayesian analysis, information on the choice of priors and Markov chain Monte Carlo settings
☒	☐	For hierarchical and complex designs, identification of the appropriate level for tests and full reporting of outcomes
☒	☐	Estimates of effect sizes (e.g. Cohen's *d*, Pearson's *r*), indicating how they were calculated

Our web collection on statistics for biologists contains articles on many of the points above.

Software and code

Policy information about availability of computer code

Data collection	Metagenomic next-generation sequencing (mNGS) data were collected on Illumina sequencers (NextSeq 550 or NextSeq 550Dx running in RUO mode) using manufacturer-provided software (NextSeq 550 Software System Suite or NextSeq 550Dx Software System Suite, versions 1 through 4). The latest version of the software was installed under an instrument service plan for maintenance and upgrades.
Data analysis	The SURPI+ (https://github.com/chiulab/SURPI-plus-dist) computational pipeline, run as a container (v1.0.0) on either a secure server or cloud infrastructure, was used for identification of pathogens from mNGS data. The steps of the SURPI+ pipeline consisted of (1) pre-processing for trimming of adapters and removal of low-complexity and low-quality reads, (2) human host background subtraction, (3) alignment to the National Center for Biotechnology Information (NCBI) GenBank NT (nucleotide) reference database for microbial identification, (4) taxonomic classification of aligned reads, and (5) visualization and interpretation of sequencing data. The SURPI+ analysis and visualization code have previously been published (Naccache et al., 2014, and Miller, et al., 2019).<br><br>Quality control (QC) metrics for the assay included a minimum of 5 million preprocessed reads per sample, >75% of data with quality score >30, and successful detection of all seven representative organisms in the positive control and the internal spiked T1 and MS2 phage controls. A threshold criterion of ≥3 non-overlapping viral reads aligning to the target viral genome was considered a positive detection for virus identification. An RPM ratio threshold of 10 was considered positive for bacteria, fungi, and parasite detection.<br><br>Statistical analyses were performed using the Python scipy package (version 1.14) as implemented in Python (version 3.12.4) or GraphPad Prism software (version 10.1.0).

For manuscripts utilizing custom algorithms or software that are central to the research but not yet described in published literature, software must be made available to editors and reviewers. We strongly encourage code deposition in a community repository (e.g. GitHub). See the Nature Portfolio guidelines for submitting code & software for further information.

Data

Policy information about availability of data

All manuscripts must include a data availability statement. This statement should provide the following information, where applicable:

- Accession codes, unique identifiers, or web links for publicly available datasets
- A description of any restrictions on data availability
- For clinical datasets or third party data, please ensure that the statement adheres to our policy

CSF mNGS results for the 4,828 samples / cases in the study are available in Supplementary Dataset 1. The Performance of CSF mNGS testing compared to other diagnostic modalities and by syndrome is available in Supplementary Dataset 2. Potentially pathogenic microbial contaminants detected in control samples by CSF mNGS testing and recorded in a pathogen contaminant database are available in Supplementary Dataset 3. Metagenomic reads from CSF samples from UCSF patients in this study were depleted of human host sequences and have been submitted to the National Center for Biotechnology Information (NCBI) BioProject database under accession number PRJNA1143941 and umbrella accession number PRJNA234047 . Please contact the corresponding author C.Y.C. regarding data access for non-UCSF patients.

Research involving human participants, their data, or biological material

Policy information about studies with human participants or human data. See also policy information about sex, gender (identity/presentation), and sexual orientation and race, ethnicity and racism.

Reporting on sex and gender	Sex reported in the medical chart (UCSF patient) or on laboratory requisition form (non UCSF patient) were used for descriptive analysis of the cohort. All other results were presented irrespective of the sex/gender. There were no sex or gender-based analyses performed because it not a relevant factor in the evaluation of a diagnostic test for central nervous system infections.
Reporting on race, ethnicity, or other socially relevant groupings	Race, ethnicity or any socially relevant groupings were not considered in this study design.
Population characteristics	The population included all patients with suspicion of central nervous system infection for whom a clinician collected a cerebrospinal fluid sample for metagenomic next-generation sequencing performed at the UCSF clinical microbiology laboratory regardless of their sex or gender. Overall, 15.2% of the cohort was less than 18 years old, 59.8% between 18 and 65 years old, and 25% over 65 years old. 35.8% of the study population was considered immunocompromised.
Recruitment	All patients for whom a metagenomic next-generation sequencing test on cerebrospinal fluid was performed by the UCSF Clinical Microbiology Laboratory were included.
Ethics oversight	Retrospective patient chart review and analysis of patient clinical CSF results were performed under a biobanking protocol (#10-01990) with waiver of consent approved by the UCSF Institutional Review Board. As the analysis was performed using CSF mNGS data from patients undergoing treatment, a waiver of consent was obtained from the UCSF IRB for both UCSF and non-UCSF patients because obtaining consent from this critically ill population of hospitalized patients with suspected central nervous system infections was not practical.

Note that full information on the approval of the study protocol must also be provided in the manuscript.

Field-specific reporting

Please select the one below that is the best fit for your research. If you are not sure, read the appropriate sections before making your selection.

☒ Life sciences ☐ Behavioural & social sciences ☐ Ecological, evolutionary & environmental sciences

For a reference copy of the document with all sections, see nature.com/documents/nr-reporting-summary-flat.pdf

Life sciences study design

All studies must disclose on these points even when the disclosure is negative.

Sample size	No sample size was calculated since all patients who received a metagenomic next-generation sequencing on cerebrospinal fluid performed at the UCSF clinical microbiology laboratory were included. This means that all available samples from the study period were considered and included in the study. The sample size is sufficient for the conclusions as this is the largest cohort published to date on CSF mNGS.
Data exclusions	No data were excluded from the analysis.
Replication	Not applicable. Results cannot be replicated because the test is done on clinical CSF samples for which there is insufficient amount to repeat testing. The CSF mNGS assay has undergone rigorous clinical validation (Miller et al., 2019) showing excellent reproducibility.
Randomization	Not applicable. All patients for whom a metagenomic next-generation sequencing test on cerebrospinal fluid was performed by the UCSF

Randomization	Clinical Microbiology Laboratory were included. Since the study is retrospective and doesn't include a control group, randomization is not possible/applicable.
Blinding	Blinding was not relevant since patients were not separated in groups. Unblinded chart-review was also necessary to collect all relevant data.

Reporting for specific materials, systems and methods

We require information from authors about some types of materials, experimental systems and methods used in many studies. Here, indicate whether each material, system or method listed is relevant to your study. If you are not sure if a list item applies to your research, read the appropriate section before selecting a response.

Materials & experimental systems

n/a	Involved in the study
☒	☐ Antibodies
☒	☐ Eukaryotic cell lines
☒	☐ Palaeontology and archaeology
☒	☐ Animals and other organisms
☒	☐ Clinical data
☒	☐ Dual use research of concern
☒	☐ Plants

Methods

n/a	Involved in the study
☒	☐ ChIP-seq
☒	☐ Flow cytometry
☒	☐ MRI-based neuroimaging

Plants

Seed stocks	Not applicable.
Novel plant genotypes	Not applicable.
Authentication	Not applicable.
